# The antiviral compound BIT225 inhibits HIV-1 replication in myeloid dendritic cells

**DOI:** 10.1186/s12981-016-0093-z

**Published:** 2016-02-08

**Authors:** Gabriela Khoury, Gary Ewart, Carolyn Luscombe, Michelle Miller, John Wilkinson

**Affiliations:** Biotron Limited, Suite 1.9, 56 Delhi Road, North Ryde, Sydney, NSW 2013 Australia

**Keywords:** HIV-1, Antiviral, Myeloid, Dendritic cells, Viral transfer

## Abstract

**Background:**

Previous studies with BIT225 (*N*-carbamimidoyl-5-(1-methyl-1*H*-pyrazol-4-yl)-2-naphthamide) have demonstrated a unique antiviral activity that blocks the release of HIV-1 from monocyte-derived macrophages (MDM). Antagonising the ion channel formed by HIV-1 Vpu, BIT225 preferentially targets de novo intracellular virus produced in ‘virus-containing compartments’ of MDM. In primary infections, dendritic cells (DC) are one of the first cells infected by HIV-1 and can transfer virus to more permissive CD4^+^ T cells, making these cells an important target for novel antiviral therapies. To extend previous findings with BIT225, we aimed to further characterise the antiviral activity of BIT225 on HIV-1 replication in monocyte-derived DC (MDDC).

**Results:**

The anti-HIV-1 activity of BIT225 was evaluated in vitro within MDDC alone and in co-cultures with activated CD4^+^ T cells to examine the effect of the drug on HIV-1 transfer. Antiviral activity was determined by measuring HIV-1 reverse transcriptase activity in the culture supernatant of BIT225 treated and DMSO control cultures. A single dose of BIT225 resulted in a mean (SE) peak inhibition of HIV-1 release from MDDC by 74.5 % (±0.6) following 14 days of culture and a 6-fold reduction of HIV-1 transfer to activated uninfected CD4^+^ T cells in co-culture.

**Conclusions:**

HIV-1 release from MDDC was inhibited by BIT225. This data broadens the drug’s antiviral activity profile within cells of the myeloid lineage. These findings suggest a potential role for BIT225 in reducing HIV-1 production and preventing viral dissemination in early and chronic infection and may assist in limiting virus spread with any ongoing viral replication during antiretroviral therapy.

## Findings

HIV-1 Vpu forms cation-selective ion channels in planar phospholipid bilayers [1] and enhances the process of virion budding and release [2]. BIT225 (*N*-carbamimidoyl-5-(1-methyl-1*H*-pyrazol-4-yl)-2-naphthamide), a novel acyl-guanidine, has been shown to inhibit the flow of ions through this viroporin [3]. Further in vitro efficacy studies of BIT225 using HIV-1 infected primary monocyte-derived macrophages (MDM), demonstrated a unique late phase inhibitory mechanism that prevents the release of virus from infected MDM [3, 4]. BIT225 has an EC_50_ of 2.25 ± 0.23 μM (mean ± SE) with minimal in vitro toxicity (TC_50_ of 284 μM) in infected MDM, resulting in a selectivity index of 126. More recently, the anti-HIV-1 activity of BIT225 towards cells of the myeloid lineage has been examined in vivo and confirms the observed activity in vitro [5]. In T cells the antiviral activity of BIT225 is ~10-fold lower and BIT225 has no inhibitory effect on HIV-2, which lacks Vpu [3]. In further support of BIT225 targeting of Vpu, the antiviral activity of BIT225 in vitro, with cell lines infected with ∆Vpu viruses, was found to be minimal [6].

Monocytes, macrophages and dendritic cells (DC) of the myeloid lineage all play important roles in establishing and maintaining HIV-1 infection in vivo. During initial infection both DC and macrophages within the vaginal and gastrointestinal tract mucosa have been shown to be one of the first cells to become infected [7–10]. These cells are able to disseminate virus to other cell types locally, setting up foci of infection [9–11] and in the case of DC, migrate to the lymph nodes and other peripheral tissues [8] within 24 h of infection [12]. DC that enter the lymph node are able to interact and transfer virus to more permissive CD4^+^ T cells resulting in high levels of virus replication [12–14] and the establishment of long-lived viral reservoirs [15].

In an extension of previous findings on the anti-HIV-1 activity of BIT225 in MDM, the objective of this study was to further characterise the effect of BIT225 on HIV-1 replication in MDDC.

## Methods

### Compound

BIT225 was prepared by dissolving stock in dimethyl sulfoxide (DMSO) and further diluted to working concentrations in culture media. BIT225 was used at a concentration of 20 µM, approximately ten times the EC_50_ [3].

### Virus

The laboratory adapted R5-tropic virus HIV-1_BaL,_ was grown and titred in HIV-1 seronegative peripheral blood mononuclear cells (PBMC) to a TCID_50_ of 8 × 10^4^ infectious doses/mL. Target cells were infected with HIV-1_BaL_ at a multiplicity of infection (MOI) of 0.04 for 3 h.

### Generation of MDDC

PBMC were isolated from seronegative donors (Australian Red Cross Blood Service) via standard Ficoll-Hypaque gradient centrifugation. Total CD14^+^ monocytes were isolated using magnetic bead depletion, as per manufacturer’s guidelines (Miltenyi Biotech, Gladbach, Germany). Isolated CD14^+^ monocytes were cultured with 7.5 ng/mL GM-CSF and 10 ng/mL IL-4 (Jomar Bioscience, SA, Australia) for 6 days in RPMI supplemented with 10 % heat inactivated foetal calf serum (FCS; Sigma-Aldrich, St Louis, MO, USA), 20 mM l-glutamine and 200 U/mL penicillin/200 µg/mL streptomycin (Sigma-Aldrich). Successful differentiation into immature MDDCs was confirmed by flow cytometry, with immature MDDC identified by the loss of CD14 expression and the expression of CD1a^+^CD4^+^DC-SIGN^+^MR^+^CD83^−^ [12].

### CD4^+^ T cell preparation

PBMC were activated for 3 days with 2.5 µg/mL phytohemmagglutinin (PHA; Sigma-Aldrich) and cultured in RPMI supplemented with 10 % heat-inactivated FCS, 20 mM L-glutamine, 200 U/mL penicillin/200 µg/mL streptomycin (Sigma-Aldrich) and 20 U/mL IL-2 (Roche Molecular Biochemicals, Indianapolis, IN). Uninfected activated CD4^+^ T cells used in viral transfer experiments, were isolated using magnetic bead selection with the CD4^+^ T cell isolation kit, as per manufacturer’s instructions (Miltenyi Biotec) to a purity >95 %.

### HIV-1 detection

Reverse transcriptase (RT) activity assay was used to quantify the amount of virus within the culture supernatants via a chemiluminescent ELISA, which detects the activity of the HIV-1 RT enzyme [16]. Inhibition of HIV-1 infection by BIT225 was determined as a percentage of the infection observed in the DMSO treated controls.

## Results

### BIT225 reduces HIV-1 production in MDDC

**Fig. 1:**
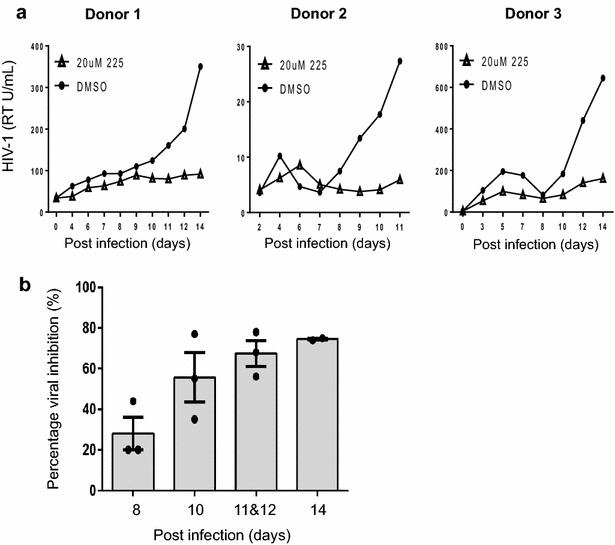
BIT225 inhibits HIV-1 replication in MDDC. MDDC were infected with HIV-1_BaL_ in the presence of 20 µM BIT225 (*open triangles*) or with equivalent DMSO solvent control (*full circles*). **a** HIV-1 replication was measured using a reverse transcriptase (RT) activity assay in three separate donors and combined as (**b)** mean (±SE) percentage viral inhibition

Day 6 immature MDDC were infected with HIV-1_BaL_ at an MOI of 0.04 for 3 h and washed three times to remove unbound virus. Infected MDDC were cultured at 1 × 10^6^ cells/mL for 14 days in the presence of 20 µM BIT225, or the equivalent DMSO solvent control, that was added once at day 0. Culture supernatants were collected periodically and HIV-1 replication determined by measuring RT activity (Fig. 1a, n = 3). Despite expected donor–donor variation in the level of HIV-1 infection of the MDDC, the antiviral activity of BIT225 was evident in all three donors compared to the DMSO controls (DMSO v BIT225 at Day 14, n = 2, p = 0.12). When represented as a mean (±SE) percentage of viral inhibition compared to the DMSO controls, the anti-HIV-1 activity of BIT225 increased over time with inhibition at 28.0 % (±87.9), 55.3 % (±12.2), 67.3 % (±11.0), and 74.5 % (±0.6) for days 8, 10, 11/12 and 14 respectively (Fig. 1b).

### BIT225 reduces HIV-1 transfer from infected MDDC to uninfected CD4^+^ T cells

To determine whether BIT225 was able to inhibit the transfer of HIV-1 from the MDDC to a more permissive CD4^+^ T cell target, infected MDDC were co-cultured with uninfected PHA-activated CD4^+^ T cells at 0, 2 and 4 h and at 1, 2, 4, 6, 7, 10, 12 and 14 days post-MDDC infection, at a ratio of 1:3 in the presence or absence of BIT225. Co-cultures were maintained for an additional 4–5 days and HIV-1 RT in the culture supernatant measured.

There are two mechanisms by which DC are able to infect CD4^+^ T cells; firstly, direct transfer of virus to CD4^+^ T cells via a virological synapse without integration of virus into the DC (in *trans*), and secondly, post-integration where de novo virus is transferred to the CD4^+^ T cell from the DC (*cis* infection) [12, 17]. HIV-1 viral burden within the intracellular compartments of the infected MDDC is degraded over time by the endolysosomal pathway, with a higher level of virus present within the MDDC at 1 h versus 8 h post-infection. The fate of the HIV-1 virion is against the clock to either transfer to a new target cell in *trans*, within 24 h, or escape the endosome and infect the DC resulting in a productive infection of the host cell [12].

**Fig. 2:**
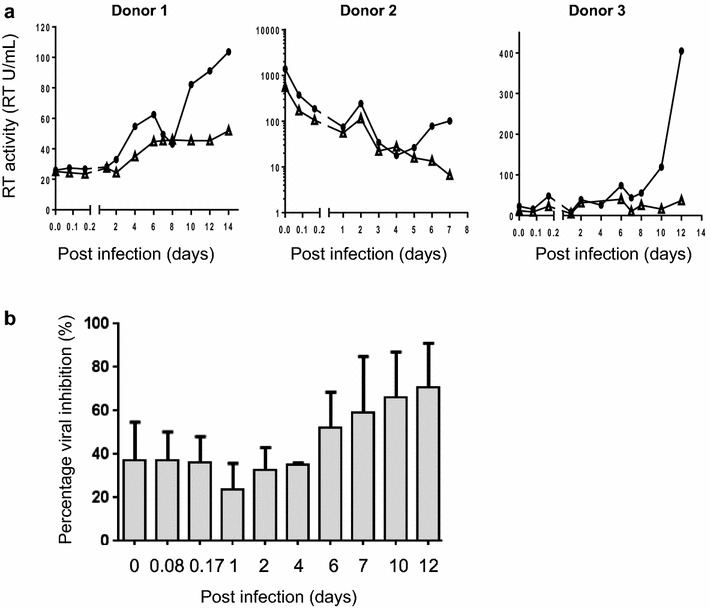
BIT225 reduces the transfer of HIV-1 from MDDC to more permissive uninfected CD4^+^ T cells. MDDC were infected with HIV-1_BaL_ in the presence of 20 µM BIT225 (*open triangles*) or with DMSO solvent control (*full circles*) and co-cultured with activated CD4^+^ T cells at various times post-infection to measure viral transfer. **a** HIV-1 replication was measured using a reverse transcriptase (RT) activity assay in three donors and data was also represented as **b** mean (±SE) percentage viral inhibition

In the co-cultures, a single BIT225 treatment of the infected MDDC resulted in a reduction in the transfer of HIV-1 from the MDDC to the uninfected CD4^+^ T cells when the source of HIV-1 was from de novo viral production, *cis* transfer (Fig. 2a). The antiviral effect of BIT225 increased over time following MDDC infection, such that increased exposure to BIT225 resulted in a decreased virus burden within the MDDC, leading to a reduction in HIV-1 transfer to the more permissive CD4^+^ T cell (DMSO v BIT225 at Day 12, n = 2, p = 0.12). When MDDC were added to the co-cultures the mean (±SE) percentage viral inhibition increased from 32.8 % (±10.3) to 70.5 % (±20.3) between days 2 and 12 post-MDDC infection (Fig. 2b).

Further assessment of viral transfer during the <24 h time points, *trans* infection, demonstrated that BIT225 treatment resulted in lower levels of HIV-1 transfer from the MDDC to the uninfected CD4^+^ T cells in the three donors (Fig. 2a). The mean (±SE) percentage inhibition of HIV-1 transfer in *trans* by BIT225, from the infected MDDC at 0, 2 and 4 h post infection, was consistent with 37 % (±17), 37 % (±13) and 36 % (±12) for these three time points (Fig. 2b).

## Discussion

Previous studies have demonstrated that BIT225 is a late phase inhibitor of HIV-1 infection in MDM with antiviral activity in vitro [3, 4] and in vivo [5]. The current study demonstrates that a single treatment with BIT225 reduces both the release of HIV-1 and the transfer of de novo virus from MDDC to activated CD4^+^ T cells targets and these effects are long-lasting.

Preventing transfer and dissemination of HIV-1 to CD4^+^ T cells has important implications for both early and late infection events. During early infection, infected DC can transmit HIV-1 to tissue resident CD4^+^ T cells at the mucosa or move from the mucosa to the lymph node where they come into contact with CD4^+^ T cells and are able to transmit the virus to these permissive target cells [9–13]. In chronic HIV-1 infection and during antiretroviral therapy, HIV-1 is detected within follicular DC within the lymph node [18, 19] where drug penetration is reduced [20]. These DC are a potential source of new viral infection of both resident follicular helper T cells [21] and circulating CD4^+^ T cells like central memory and naïve CD4^+^ T cells [22]. BIT225, as a drug with preferential anti-HIV-1 activity in cells of the myeloid lineage, may be beneficial in targeting ongoing HIV-1 persistence. Although BIT225 can cross the blood–brain barrier [5], the ability of BIT225 to penetrate other drug sanctuary sites to effective levels, such as the lymph nodes, is currently unknown.

In summary, the additional data reported here which further characterise the anti-HIV-1 effect of BIT225 demonstrate that this compound is able to inhibit the release of HIV-1 from MDDC and in turn reduce the transfer of virus from these cells to uninfected CD4^+^ T cells in co-culture. BIT225 has the potential to play an important role in preventing the dissemination of virus at both early and late stages of infection, limit the establishment of long-lived viral reservoirs in cells of the myeloid lineage and potentially prevent reseeding of the reservoir.

## Abbreviations

CD: cluster of differentiation
DC: dendritic cell
DMSO: dimethyl sulfoxide
EC_50_: half maximal effective concentration
ELISA: enzyme-linked immunosorbent assay
FCS: foetal calf serum
GLP: good laboratory practice
GM-CSF: granulocyte-macrophage colony-stimulating factor
HIV-1/2: human immunodeficiency virus type 1/2
IL: interleukin
MDDC: monocyte-derived dendritic cell
MDM: monocyte-derived macrophage
MOI: multiplicity of infection
M: molar
PBMC: peripheral blood mononuclear cells
PHA: phytohaemagglutinin
RT: reverse transcriptase
SE: standard error
TCID_50_: tissue culture infectious dose (resulting in a 50 % response)
U: units
Vpu: viral protein unique
